# Activity-dependent modulation of inhibitory synaptic kinetics in the cochlear nucleus

**DOI:** 10.3389/fncir.2014.00145

**Published:** 2014-12-23

**Authors:** Jana Nerlich, Christian Keine, Rudolf Rübsamen, R. Michael Burger, Ivan Milenkovic

**Affiliations:** ^1^Department of Neurobiology, Faculty of Biosciences, Pharmacy and Psychology, University of LeipzigLeipzig, Germany; ^2^Department of Biological Sciences, Lehigh UniversityBethlehem, PA, USA; ^3^Department of Physiology, Faculty of Medicine, Carl Ludwig Institute for Physiology, University of LeipzigLeipzig, Germany

**Keywords:** inhibition, activity-dependent decay, re-uptake, intersynaptic pooling, asynchronous release, spherical bushy cell, cochlear nucleus

## Abstract

Spherical bushy cells (SBCs) in the anteroventral cochlear nucleus respond to acoustic stimulation with discharges that precisely encode the phase of low-frequency sound. The accuracy of spiking is crucial for sound localization and speech perception. Compared to the auditory nerve input, temporal precision of SBC spiking is improved through the engagement of acoustically evoked inhibition. Recently, the inhibition was shown to be less precise than previously understood. It shifts from predominantly glycinergic to synergistic GABA/glycine transmission in an activity-dependent manner. Concurrently, the inhibition attains a tonic character through temporal summation. The present study provides a comprehensive understanding of the mechanisms underlying this slow inhibitory input. We performed whole-cell voltage clamp recordings on SBCs from juvenile Mongolian gerbils and recorded evoked inhibitory postsynaptic currents (IPSCs) at physiological rates. The data reveal activity-dependent IPSC kinetics, i.e., the decay is slowed with increased input rates or recruitment. Lowering the release probability yielded faster decay kinetics of the single- and short train-IPSCs at 100 Hz, suggesting that transmitter quantity plays an important role in controlling the decay. Slow transmitter clearance from the synaptic cleft caused prolonged receptor binding and, in the case of glycine, spillover to nearby synapses. The GABAergic component prolonged the decay by contributing to the asynchronous vesicle release depending on the input rate. Hence, the different factors controlling the amount of transmitters in the synapse jointly slow the inhibition during physiologically relevant activity. Taken together, the slow time course is predominantly determined by the receptor kinetics and transmitter clearance during short stimuli, whereas long duration or high frequency stimulation additionally engage asynchronous release to prolong IPSCs.

## Introduction

Synaptic inhibition is mainly mediated by glycine and GABA_A_ receptors (GlyR and GABA_A_R, respectively) which tightly regulate neuronal and network activities. While the GlyR-generated inhibitory postsynaptic current (IPSC) is generally endowed with fast decay kinetics (Takahashi et al., 1992; Awatramani et al., 2004), GABA provides slow and in some cases tonic inhibition (Farrant and Nusser, 2005; Capogna and Pearce, 2011; Tang et al., 2011). Notably, there are presynaptic terminals in the sensory systems (Wentzel et al., 1993; Protti et al., 1997; Apostolides and Trussell, 2014), cerebellum (Dumoulin et al., 2001; Rousseau et al., 2012; Husson et al., 2014) and in the spinal cord (Jonas et al., 1998; O’Brien and Berger, 1999; Keller et al., 2001; Seddik et al., 2007) that release both transmitters beyond the early postnatal development. This allows for an additional variability, through activity-dependent use of transmitters (Nerlich et al., 2014), differential distribution of respective receptors at the same cell (Chéry and de Koninck, 1999), at different cells (Dugué et al., 2005; Kuo et al., 2009), or shaping the IPSC decay through action of both glycine and GABA on GlyR (Lu et al., 2008).

The postsynaptic responses in neurons expressing both GlyR and GABA_A_R usually represent a mixture of respective fast and slow synaptic currents (Russier et al., 2002; Awatramani et al., 2005; González-Forero and Alvarez, 2005; Coleman et al., 2011; Apostolides and Trussell, 2013). The relative contribution of both physiologically important components can change according to the activity pattern (Fischl et al., 2014). Such activity-dependent inhibitory control, mediated by dual glycine-GABA signaling, has been recently shown for the spherical bushy cells (SBCs) in the central auditory system (Nerlich et al., 2014). SBCs receive acoustically evoked excitatory input from auditory nerve fibers through large calyceal terminals, the endbulds of Held (Ryugo and Sento, 1991; Isaacson and Walmsley, 1996; Nicol and Walmsley, 2002), and non-primary inhibition from neurons within the cochlear nucleus (Wickesberg and Oertel, 1990; Saint Marie et al., 1991; Campagnola and Manis, 2014). The amplitude of IPSCs is dominated by GlyRs, while the initially small GABAergic component successively enhances the inhibitory strength and shapes its duration at physiologically relevant rates (Nerlich et al., 2014). Unlike other central auditory synapses that utilize phasic inhibition even at high rates (Awatramani et al., 2004; Kramer et al., 2014), IPSCs in SBCs summate due to slow kinetics (Xie and Manis, 2013, 2014; Nerlich et al., 2014), thereby providing a functionally tonic inhibition, similar to inhibition acting on the granule cells in the dorsal cochlear nucleus (DCN) and nucleus magnocellularis neurons in the cochlear nucleus of the chick (Lu and Trussell, 2000; Monsivais et al., 2000; Balakrishnan et al., 2009). To date, the synaptic mechanisms that determine the slow kinetics of the mixed glycine-GABA transmission in SBCs remained elusive.

We examined the mechanisms underlying the slow activity-dependent IPSC kinetics in SBCs by performing whole-cell recordings in acute slice preparations of juvenile Mongolian gerbils in combination with synaptic stimulation of inhibitory inputs. Our results demonstrate that the low capacity of glycine and GABA uptake allows transmitter rebinding, particularly at input rates above 100 Hz. The activity-driven transmitter spillover possibly engaged distant GlyR but not GABA_A_R. Synaptic activity largely desynchronized the release of glycine and GABA, which had a significant contribution to the slow IPSC profile.

## Materials and methods

The experimental procedures were approved by the Saxonian district Government Leipzig (T 84/12, T 67/13) and conducted according to the European Communities Council Directive (86/609/EEC).

### Slice preparation

Coronal slices (180 μm) containing the rostral anteroventral cochlear nucleus (AVCN) were cut from P22-P30 gerbils of either sex. The brainstem was sliced with a vibratome in low-calcium artificial cerebrospinal fluid (ACSF) solution containing (in mM): 125 NaCl, 2.5 KCl, 0.1 CaCl_2_, 3 MgCl_2_, 1.25 NaH_2_PO_4_, 25 NaHCO_3_, 25 glucose, 2 sodium pyruvate, 3 myo-inositol, 0.5 ascorbic acid, continuously bubbled with 5% CO_2_ and 95% O_2_, pH 7.4. Slices were incubated in the standard recording solution (ACSF same as for slicing, except CaCl_2_ and MgCl_2_ were changed to 2 mM and 1 mM, respectively) for 30 min at 37°C and stored at room temperature until recording. Experiments were performed at nearly physiological temperature (33.5 ± 0.5°C).

### Electrophysiological recordings

Whole-cell patch clamp recordings were performed on SBCs in the rostral pole of the AVCN. Due to their large soma size and localization in the low-frequency area of the gerbil AVCN, these neurons can be visually distinguished from globular bushy cells. Morphological verification of SBCs was done during the recording by intracellular labeling with ATTO 488 and visualization by a CCD camera (IMAGO Typ VGA; TILL Photonics). The pipettes had resistances of 3–4 MΩ when filled with (mM): 125 CsMeSO_3_, 18 TEA-Cl, 3 MgCl_2_, 10 HEPES, 0.1 EGTA, 4.5 QX-314-Cl, 5 phosphocreatine, 2 ATP disodium salt, 0.3 GTP disodium salt, and 50 μM ATTO 488 (pH 7.3 with CsOH). The resulting inward currents with larger amplitudes enabled more accurate analyses compared to the small events occurring with physiological [Cl^−^]_pip_. Consistent with slow inhibitory kinetics in the present study, the synaptically evoked hyperpolarizations acquired with gramicidin perforated patch recordings also exhibited slow activity-dependent synaptic decays (gramicidin perforated patch: t_wd_ single: 18.5 ± 4.0 ms, 100 Hz 10th IPSC: 42.6 ± 4.1 ms, *n* = 6, see Nerlich et al., 2014). IPSCs were evoked by electrical stimulation of afferent fibers through a bipolar theta glass electrode (Science Products, tip Ø 5 μm) filled with bath solution and placed at distances of 30–60 μm from the cell. Pulse stimuli (100 μs, 15–90 V) were generated by a stimulator (Master 8) and delivered via a stimulus isolation unit (AMPI Iso-flex) to evoke either single events or train-responses at different frequencies. Voltage clamp measurements were done from V_hold_ = −71 mV, (Nerlich et al., 2014), except in cases where the amplitudes of the small asynchronous events were increased for precise event detection by holding the cell at −81 mV (Figure 5). To isolate glycine- and GABA_A_ receptor mediated signals, a pharmacological inhibition of glutamate (50 μM AP-5, 10 μM NBQX) and GABA_B_receptors (3 μM CGP 55845) was performed in all experiments. Offline correction of voltages was done for junction potentials of 11 mV. For some experiments, the extracellular calcium concentration in the ACSF was reduced to 1.2 mM in order to decrease the release probability. In such cases, the magnesium concentration was simultaneously increased to 1.8 mM to maintain the concentration of divalent cations.

**Figure 1:**
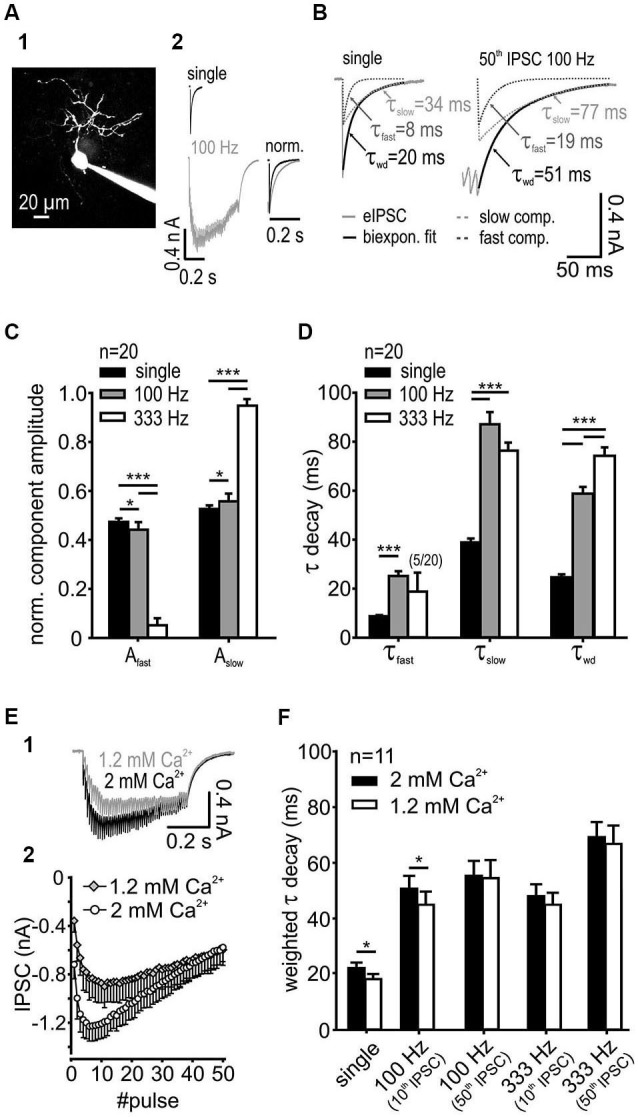
**IPSC decay kinetics are activity-dependent. (A1)** SBC labeled with ATTO 488 reveals its characteristic morphology. **(A2)** Exemplary IPSCs elicited by single and 100 Hz stimulation of synaptic inputs. Right: Superimposed normalized IPSC from single-pulse, and 50-pulse responses, the latter showing decay times prolonged by train stimulation. **(B)** IPSCs decay was best fit with a bi-exponential function with the decay time constants τ_fast_ and τ_slow_; also shown are the respective weighted decay time constants τ_wd_ **(C)** The respective component amplitudes A_fast_ and A_slow_ varied systematically with stimulation frequency. The fraction of the slow exponential component increased at higher input frequencies (*n* = 20, *p < 0.05, ***p < 0.001, RM ANOVA) with a complementary decrease in the fast component amplitude. **(D)** Fast and slow decay time constants at different input frequencies (*n* = 20). Compared to single pulse stimulation, the increased input rate prolonged τ_fast_ at 100 Hz (*p* < 0.001, paired *t*-test) and τ_slow_ at both 100 Hz and 333 Hz (*p* < 0.001, RM ANOVA). Activity-dependent change of the τ_fast_ and τ_slow_ and the respective amplitudes at 100 Hz and 333 Hz (see panel **C**) resulted in a prolonged τ_wd_ of repetitive IPSCs (*p* < 0.001, RM ANOVA). **(E)** The release probability determines the temporal profile and the inhibitory strength at the onset of an IPSC train. ** (E1)** Traces of IPSCs under standard (2 mM, used for slice recordings) and reduced (1.2 mM) extracellular calcium concentration.** (E2)** Mean baseline amplitudes of 50 IPSCs in a 100 Hz train under different extracellular calcium concentrations (*n* = 6). **(F)** For single IPSCs and the 10th IPSC at 100 Hz the weighted decay time constant was shorter under 1.2 mM extracellular calcium. [Ca^2+^]*_o_* had no influence on τ_wd_ at longer trains of IPSCs (50 pulses) or at higher input frequency (333 Hz) (*n* = 11, single and 100 Hz 10th *p* < 0.05, 100 Hz 50th, 333 Hz 10th and 50th, *p* > 0.27, RM ANOVA).

**Figure 2:**
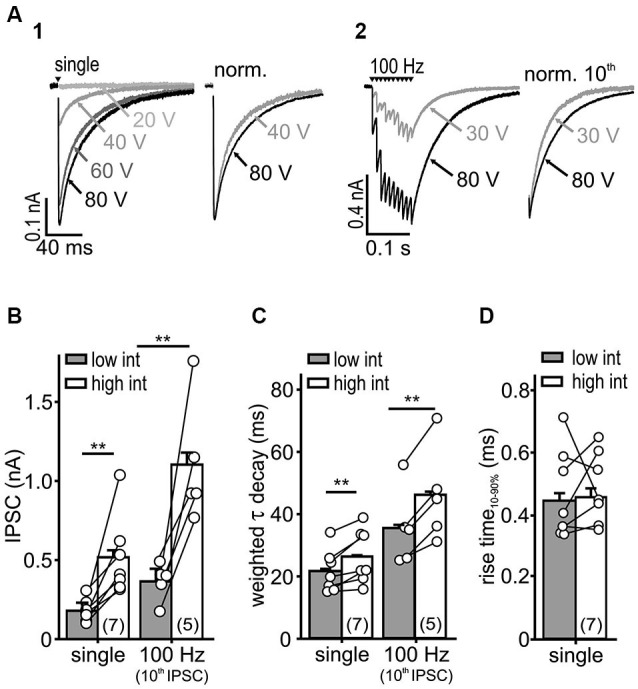
**IPSC kinetics depends on stimulus intensity. (A)** Representative IPSC traces evoked by a single **(A1)** or repetitive stimulation **(A2)** at varying stimulus intensities. Higher stimulus intensities evoked IPSCs of longer decays during single and repetitive stimulations (right: normalized last IPSCs). **(B,C)** Both IPSC amplitude **(B)** and τ_wd_ **(C)** increased with stimulation intensity (cell numbers are given in parentheses, *p* < 0.01, paired *t*-test). **(D)** The IPSC rise-time was unaffected by the stimulus intensity (*n* = 7, *p* = 0.79, paired *t*-test).

**Figure 3:**
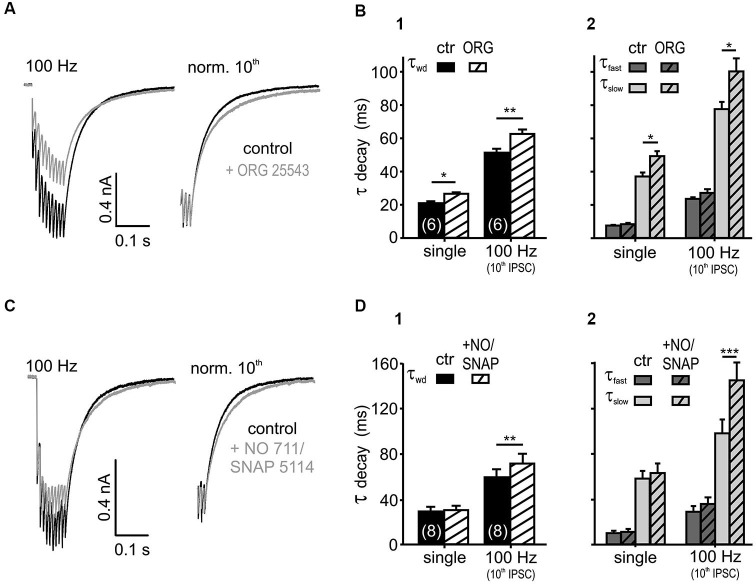
**Glycine and GABA re-uptake contributes to the IPSC kinetics. (A)** Average traces of 100 Hz IPSC trains, before and after the bath application of the GlyT2 blocker ORG 25543 (20 μM). The weighted time constant increased under the glycine re-uptake block (right: traces normalized to the last IPSC). **(B)** Inhibition of GlyT2 prolonged the τ_wd_ **(B1)** upon single (*p* < 0.05) and repetitive (*p* < 0.01) synaptic stimulation due to an increase in τ_slow_ (*p* < 0.05). The fast exponential component was unaffected **(B2)** (*n* = 6, *p* = 0.15, RM ANOVA). **(C)** IPSCs evoked at 100 Hz, before and after the bath application of 20 μM NO 711 and 100 μM SNAP 5114. The weighted decay time constant increased under the GABA re-uptake block (right: traces normalized to the last IPSC). **(D)** Inhibition of GABA uptake prolonged the weighted decay of IPSCs at 100 Hz (*p* < 0.01) **(D1)** by increasing τ_slow_ (*p* < 0.001) **(D2)**. The IPSC kinetics of single events were not affected (*p* > 0.45, *n* = 8, RM ANOVA).

**Figure 4:**
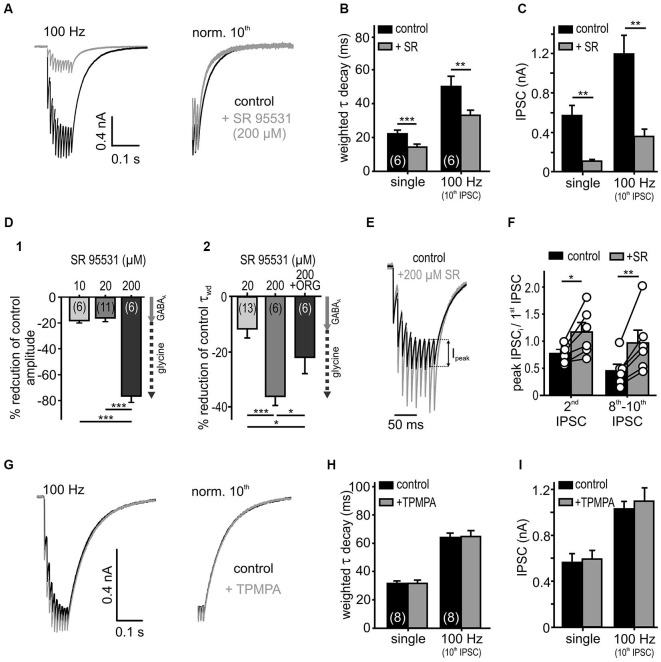
**Low affinity antagonist reveals spillover and rebinding of glycine. (A)** Responses to 10 stimuli at 100 Hz, under the control condition and in the presence of a weak glycine receptor antagonist SR 95531 (200 μM). The weighted decay decreased under SR 95531 (right: traces normalized to the last IPSC). **(B,C)** Summary data showing faster IPSC decay time constants **(B)** and substantially reduced amplitudes **(C)** under the weak antagonism of SR95531 for single and 100 Hz stimulation (*n* = 6, RM ANOVA). **(D)** Percentage reduction of amplitudes **(D1)** and decay values **(D2)** of single IPSCs under different SR 95531 concentrations and SR 95531+ ORG 25543 compared to control condition (100%). At concentrations of 10 μM and 20 μM, SR 95531 had similar inhibitory effects on IPSC amplitude, due to a specific GABA_A_R antagonism (Nerlich et al., 2014) (gray arrow = GABA_A_ receptor contribution). At a concentration of 200 μM, SR 95531 further reduced the amplitude and accelerated the IPSCs decay time. The faster IPSCs under 200 μM SR 95531, were again prolonged after the additional inhibition of GlyT2 by ORG 25543 (20 μM) **(D2)** suggesting a low-affinity inhibition of glycine receptors by 200 μM SR 95531 (dashed arrow = putative glycine receptor blockade). Cell numbers are given in parentheses; ANOVA. **(E)** Representative traces (10 IPSCs at 100 Hz) for control condition and superfusion of 200 μM SR 95531 normalized to the first event. I_peak_: difference between steady state of the preceding event and peak of the successive event. **(F)** The peak amplitude of the second IPSC and the mean of the 8th–10th peak IPSCs relative to the first IPSC increased in the presence of SR 95531 (2nd/1st IPSC ratio increased by 0.46 ± 0.12, *p* < 0.05; 8th–10th/1st IPSC ratio increased by 0.96 ± 0.23, *p* < 0.01, paired *t*-test, symbols: single cell data, bars: mean ± SEM). The low affinity antagonism of GlyR counteracts depression. **(G)** Low affinity GABA_A_R antagonist (200 μM TPMPA) had no effect on IPSCs (normalized to the 10th IPSC: right). **(H,I)** Summary data showing a lack of TPMPA effect on the IPSC decays (single: *p* = 0.96, 100 Hz: *p* = 0.62) and the amplitudes (single: *p* = 0.66, 100 Hz: *p* = 0.19, *n* = 8, RM ANOVA). *p < 0.05, **p < 0.01, ***p < 0.001.

**Figure 5:**
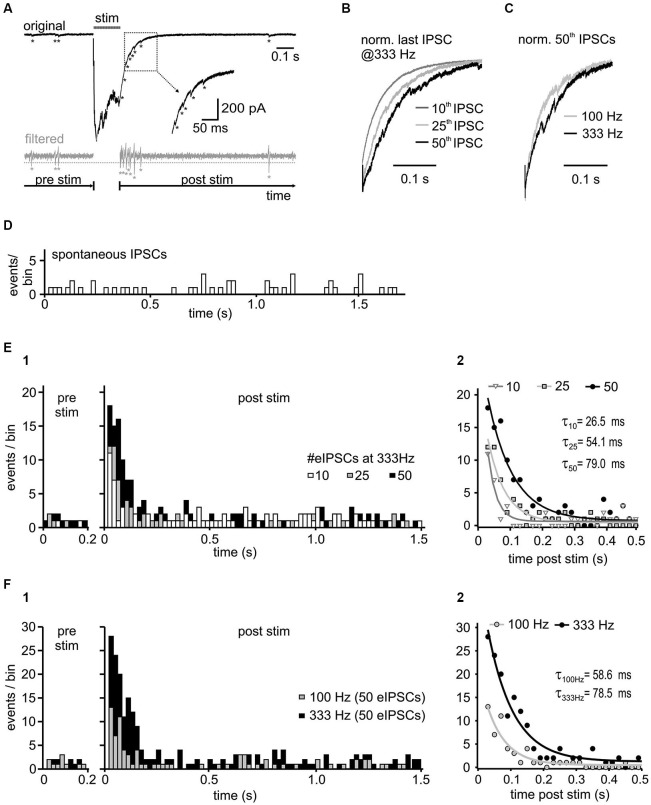
**Activity-dependence of asynchronous release. (A)** Representative trace with detected asynchronous release events, before and after 50 stimuli at 333 Hz (upper panel). For event detection the signal was bandpass filtered between 50 and 1500 Hz (bottom panel). Potential events were detected when the filtered current amplitude exceeded 1.96 times (*p* < 0.05) the standard deviation of the noise (dashed gray line). Note the increased incidence of events in the decay phase of the last IPSC. **(B)** Normalized last IPSCs elicited by different numbers of stimuli in a 333 Hz train. **(C)** Superimposed normalized last IPSCs in a 50-pulse train at 100 Hz and 333 Hz. **(D)** Histogram of spontaneous IPSCs before synaptic stimulation of inhibitory inputs (*n* = 4 cells, bin width: 20 ms). **(E1)** Histograms of asynchronous release events before ( = spontaneous events, pre stim) and after the 333 Hz input stimulation ( = asynchronous and spontaneous events, post stim) with different number of stimuli (*n* = 4 cells, bin width: 20 ms, post stim *t* = 0 refers to the time of the last IPSC in the train). Following synaptic stimulation, the time course of asynchronous events relaxed back to the resting state with a mono-exponential function. The increase of asynchronous events during the decay phase depends on the presynaptic activity. **(E2)** Mono-exponential fits to the data from **(E1)**. The initial incidence of events, and the time constant (τ) depend on stimulus number (10 vs. 25 vs. 50 stimuli *p* < 0.05 for each comparison). **(F)** Input frequency determines the amount of asynchronous release.** (F1)** Summary histograms of asynchronous events from 6 cells, before and after 50-pulse stimulation at 100 Hz and 333 Hz. **(F2)** Mono-exponential fits to the data from F1. The initial incidence of events was increased and the exponential time constant prolonged with higher input frequency (100 Hz vs. 333 Hz *p* < 0.05).

### Data aquisition and analysis

The recordings were acquired using a Multiclamp 700B amplifier (Molecular Devices). The mean capacitance of the cells was 23.15 ± 4.34 pF (mean ± SD, *n* = 52). The average series resistance was 11.25 ± 1.56 MΩ (*n* = 52), which was compensated by 50% to a remaining Rs of 3–7 MΩ. During experiments the series resistance changed on average by 2.6% (*n* = 52). Cells with series resistance changes >10% were excluded from analysis. There was no correlation between the IPCS amplitudes and decay time constants (*r*_s_ = 0.17, *p* = 0.22, *n* = 53). Together with the previously reported lack of correlation between the IPSC amplitudes and rise times (*r*_s_ = 0.04, *p* = 0.78, *n* = 43, Nerlich et al., 2014), these data rule out the possible contribution of series resistance error to decay time constant measurements. Recorded signals were digitized at 50 kHz and filtered with a 6 kHz Bessel low-pass filter. Data were examined with pClamp 10 software (Molecular Devices) followed by analyses using custom-written Matlab routines (version 8.3, Mathworks, Natick). Mean amplitudes, 10–90% rise times and decay time constants of IPSCs were analyzed from averaged traces (>5 repetitions). For detection of asynchronous release events, the current traces were band-pass filtered between 50 and 1500 Hz using a zero-phase forward and reverse digital IIR filter to remove the decay component (see Figure 5A). Statistical analysis by means of *z*-test revealed events with current amplitudes exceeding 1.96 times (*p* < 0.05) the standard deviation of the baseline. To avoid multiple triggers of the same event, all triggers occurring within 5 ms after the preceding event were excluded from the analysis. The results were visually controlled to ensure the correct detection of spontaneous events. The events occurring prior to electrical stimulation (spontaneous IPSCs) were quantified and compared to events emerging after the end of synaptic stimulation of inhibitory inputs. Asynchronous events were clearly visible during the decay phase of the last evoked IPSC. Events detected by the software during the first 20 ms of the current decay were not included into the analysis to avoid false positive results. Data from 3 repetitions for each condition were pooled and presented for each cell.

IPSC decay phase was fitted with mono- or bi-exponential functions based on an increase in adjusted R^2^ values. The weighted τ decay was calculated as τ_wd_ = (A_fast_ × τ_fast_ + A_slow_ × τ_slow_ )/(A_fast_ + A_slow_), where A_fast_ and A_slow_ are amplitudes at *t* = 0 and τ_fast_ and τ_slow_ are the fast and slow time constants, respectively. In cases of mono-exponential fits, one exponential component was set to 0.

### Statistics

Data sets were compared with the appropriate *t-*test or analysis of variance (ANOVA). The *p*-values of multiple comparisons were adjusted by the Dunn-Šidak procedure. Within-subject comparisons were performed by repeated-measures (RM) ANOVA after testing for sphericity using Mauchly test, and Greenhouse-Geissner correction applied as appropriate. ANOVA was used to test for effects of drugs and possible interactions of input frequency and drug superfusion. The changes in asynchronous release evoked by different input frequencies were tested for significance by a *z*-test, *z* = (A−BL)/SD*_BL_*, with A being the average number of events/bin 20–60 ms after stimulation, BL the mean of the baseline (1 s prior to stimulation = spontaneous IPSC level) and SD*_BL_* the standard deviation of the baseline. All data are reported as mean ± SEM, unless otherwise stated.

## Results

### IPSC decay rate is activity-dependent

SBCs exhibit slow inhibitory synaptic decays (Figure 1A) mediated by GlyR and GABA_A_R (gerbil: spontaneous IPSCs: τ_wd_ = 16.7 ± 1.2 ms, synaptically evoked IPSCs τ_wd_ = 23.7 ± 5.3 ms, Nerlich et al., 2014; mouse: spontaneous IPSCs: τ_wd_ = 8.75 ± 0.6 ms, synaptically evoked IPSCs τ_wd_ = 11.1 ± 0.8 ms, Xie and Manis, 2013). Both the glycinergic and the GABAergic components were observed in all pharmacology experiments acquiring IPSCs with suprathreshold stimulation to evoke reliable responses (see also Nerlich et al., 2014). Notably, the pharmacologically isolated glycinergic and GABAergic single events were previously shown to have similar kinetics (Nerlich et al., 2014), thus arguing against the hypothesis that in SBCs the fast decay component can be accounted to glycine and the slow component to GABA. In the present study we investigated the underlying mechanism determining such inhibitory current kinetics. The decay phase of synaptically evoked single IPSCs was best fit with a bi-exponential function with a fast decay time constant τ_fast_ = 8.7 ± 2.1 ms, a slow decay time constant τ_slow_ = 38.9 ± 7.2 ms and their respective relative amplitudes A_fast_ = 0.47 ± 0.06 and A_slow_ = 0.53 ± 0.06. The resulting average weighted decay time constant for 20 SBCs was τ_wd_ = 24.7 ± 5.4 ms (mean ± SD). The synaptic current decays were progressively slower after repetitive stimulation of inputs and with increasing input frequencies (Figures 1B,D). Also, the relative amplitude of the slow exponential component increased with input rates (Figure 1C; A_slow_ single = 0.53 ± 0.06, 100 Hz = 0.56 ± 0.03; 333 Hz = 0.95 ± 0.03, single vs. 100 Hz *p* < 0.05; 100 Hz vs. 333 Hz *p* < 0.001, *n* = 20, RM ANOVA). Complementary decrease in amplitude of the fast component occurred over the same range of stimuli (A_fast_ single = 0.47 ± 0.06, 100 Hz = 0.44 ± 0.03, 333 Hz = 0.05 ± 0.03). The slow decay time constant was prolonged substantially at 100 Hz and 333 Hz (Figure 1D; τ_slow_ single = 38.9 ± 1.6 ms, 100 Hz = 87.1 ± 5.0 ms, 333 Hz = 76.3 ± 3.3 ms, *n* = 20, *p* < 0.001 for single vs. 100 Hz and 333 Hz, RM ANOVA). At 333 Hz, the decay phase was best fit with a mono-exponential function excluding the fast exponential component in 75% of recorded SBCs (in these cells A_fast_ was set to 0). Due to the rare occurrence of τ_fast_ at 333 Hz (only in 5 out of 20 cells), this parameter was statistically compared only for the single and 100 Hz stimulation. The τ_fast_ was prolonged at 100 Hz compared to single pulse stimulation (τ_fast_ single = 7.1 ± 0.6, 100 Hz = 21.4 ± 3.0, *n* = 20, single vs. 100 Hz *p* < 0.001, paired *t*-test). Activity-dependent change of τ_fast_, τ_slow_ and the respective amplitudes resulted in a prolongation of τ_wd_ with increasing input frequency (Figure 1D; τ_wd_ single = 24.7 ± 1.2 ms, 100 Hz = 58.8 ± 2.8 ms, 333 Hz = 74.2 ± 3.5 ms, *n* = 20, *p* < 0.001 for all comparisons, RM ANOVA). However, the longer decays were not associated with larger IPSC amplitudes (τ_wd_ single = 22.8 ± 0.8 ms, 50th 100 Hz = 61.8 ± 2.5 ms, *n* = 25, *p* < 0.001, paired *t*-test; IPSC amplitude single = 0.56 ± 0.5 nA, 50th 100 Hz = 0.55 ± 0.04 nA, *n* = 25, *p* = 0.81, paired *t*-test), thus suggesting that activity-dependent synaptic mechanisms, rather than series resistance errors cause the decay time prolongation. Together, these data indicate that the increase in input rates increases the transmitter quantity in the cleft which particularly prolongs the τ_slow_ through transmitter rebinding.

Studies at the calyx of Held-MNTB principal neuron synapse revealed that *in vivo* release probability may be approximated in slice recordings by using 1.2 mM Ca^2+^ in the extracellular solution (Borst, 2010). The dependance of the IPSC decay times on the release probability was shown at other auditory synapses (Balakrishnan et al., 2009; Tang and Lu, 2012). Although the *in vivo* release probability for the endbulb of Held-SBC synapse remains to be elucidated, we still addressed the question, whether the slow decay of inhibitory currents measured in SBCs is due to an increased release probability in the slice recordings. For this, the weighted decay time constant was measured from the last event within trains consisting of different numbers of pulses and the results were compared for 1.2 mM and 2 mM external calcium concentration (Figure 1F). Single IPSCs and short trains (10 pulses at 100 Hz) revealed faster decay time constants under the lower release probability condition. However, with increasing stimulus number and frequency, the weighted decay time constant of the last pulse in the train was similar between the two calcium conditions, (Figure 1F; main effect calcium *p* = 0.04, 1.2 vs. 2 mM: single, 100 Hz 10th IPSC *p* < 0.05, 100 Hz 50th IPSC *p* = 0.72, 333 Hz 10th *p* = 0.27 and 50th IPSC *p* = 0.33, *n* = 11, RM ANOVA). The slower IPSC at the higher release probability could either be due to transmitter pooling or to multivesicular release. The latter mechanism could explain the data for the single and short IPSC trains. The reduction of extracellular calcium to 1.2 mM decreased the IPSCs amplitudes, especially at the onset of a 100 Hz train (50 pulses) (Figure 1E). However, at the end of the train the baseline IPSC amplitudes were similar to those measured under 2 mM [Ca^2+^]*_o_* (IPSC amplitudes 2 mM vs. 1.2 mM: onset = 1st IPSC, *p* < 0.001; maximum = mean 6–8th IPSC vs. mean 10–12th IPSC, *p* < 0.01; end = mean 48–50th IPSC, *p* = 0.88, *n* = 6, RM ANOVA). Thus, the release probability is unlikely to play a role for longer trains of stimuli or high frequency stimulation pointing to transmitter pooling as a mechanism contributing to slow decays.

### Recruitment of fibers slows the IPSC decay

Recruitment of nearby inputs can lead to spillover of transmitter to remote synaptic or extrasynaptic sites, and thereby to intersynaptic pooling. Thus, it is conceivable that the slow IPSC decay in SBCs is caused by ongoing rebinding of transmitter and activation of extrasynaptic receptors, as shown earlier (Balakrishnan et al., 2009; Tang and Lu, 2012). To determine whether synaptic recruitment influences the kinetics of inhibition in SBCs, IPSCs were evoked with increasing stimulus intensities (Figure 2A). The low intensity was set slightly above the threshold with IPSC amplitudes reaching 38.5 ± 16% (mean ± SD, *n* = 7) of the maximal amplitude evoked by high stimulation intensity. With an increase in stimulus intensity, the amplitude of a single evoked IPSC and the last IPSC in a 100 Hz train increased threefold (Figure 2B; low vs. high intensity: single, *n* = 7, *p* < 0.01; 100 Hz 10th IPSC, *n* = 5, *p* < 0.01, paired *t*-test). Furthermore, the weighted decay time constants of single and repetitive IPSCs were prolonged at high stimulus intensities (Figure 2C; τ_wd_ single: low = 21.6 ± 0.6 ms, high = 26.3 ± 0.6 ms, *n* = 7, *p* < 0.01, 100 Hz 10th IPSC: low = 35.4 ± 0.8 ms, high = 46.0 ± 0.8 ms, *n* = 5, *p* < 0.01, paired *t*-tests). On the other hand, the rise time of single IPSCs was not dependent on stimulus intensity, suggesting the recruitment of nearby synaptic inputs (Figure 2D; low = 0.44 ± 0.03 ms, high = 0.46 ± 0.03 ms, *n* = 7, *p* = 0.79, paired *t*-test). As we neither observed a build-up, nor a run-down of IPSC amplitudes during repetitions at a given stimulus intensity, it is unlikely that the number of activated inputs changed. Therefore, these data suggest that the transmitter quantity substantially contributes to slow inhibitory kinetics.

### Transmitter uptake contributes to the IPSC kinetics

If transmitter spillover and its clearance from the synaptic cleft shape the IPSC decay in an activity-dependent manner, the postsynaptic currents should be sensitive to a blockade of re-uptake transporters (Otis et al., 1996; Balakrishnan et al., 2009; Tang and Lu, 2012). Bath application of the glycine transporter 2 (GlyT2) inhibitor ORG 25543 (20 μM, Bradaïa et al., 2004; Balakrishnan et al., 2009) slowed down the decay of both single and train IPSCs (Figures 3A,B; *n* = 6, RM ANOVA). The fast decay time constant, the respective amplitudes of the exponential components and the IPSC rise time were not affected by the glycine re-uptake block (*n* = 6, control vs. +ORG, τ_fast_: *p* = 0.15; A_slow_ and A_fast_: *p* = 0.37, RM ANOVA, rise time: *p* = 0.99, paired *t*-test). Therefore, it can be concluded that the τ_wd_ prolongation (Figure 3B) is caused by the significantly longer τ_slow_ (Figure 3B). Given the similar IPSC decays of the isolated glycinergic and GABAergic single events (Nerlich et al., 2014), the τ_fast_ probably describes the intrinsic kinetics of receptor activation and de-activation, whereas the transmitter clearance, relief from saturation and delayed release determine τ_slow_.

In addition to the sensitivity to glycine re-uptake, the IPSC decay phase was also shaped by GABA clearance. Simultaneous inhibition of the respective neuronal- and glial-GABA transporters with 20 μM NO 711 (Szabadics et al., 2007; Tang and Lu, 2012) and 100 μM SNAP 5114 (Keros and Hablitz, 2005; Song et al., 2013) extended the weighted decay time constant of the last IPSC in 100 Hz trains. Again, the prolongation of τ_slow_ during re-uptake blockade underlie the slowing of train IPSCs (Figures 3C,D; *n* = 8, control vs. +NO/SNAP, τ_slow_: *p* < 0.001, τ_wd_: *p* < 0.01, RM ANOVA). Notably, the GABA uptake is apparently only contributing to the decay during ongoing activity, as single evoked IPSC decays were not affected (*p* > 0.45) (Figure 3D). Similar to the effects seen after blockade of glycine re-uptake, GABA re-uptake inhibition did not change the fast decay time constants, the respective amplitudes of the exponential components and the IPSC rise times (*n* = 8, control vs. +NO/SNAP, τ_fast_: *p* = 0.11, A_slow_ and A_fast_: *p* = 0.43, RM ANOVA, rise time: *p* = 0.40, paired *t*-test). Together, these data suggest that glycine rebinding caused by transmitter pooling due to slow clearance is an important factor in shaping the inhibitory current profile. On the other hand, the GABA re-uptake only contributes at high stimulus input rates to the decay kinetics, when it presumably accumulates in the synaptic cleft.

To investigate transmitter rebinding and the possible contribution of spillover to inhibitory kinetics, we assessed the effects of low affinity GlyR and GABA_A_R antagonists. The rationale to use weak competitive antagonists to probe for putative remote synapses arises from studies showing that the receptors distant to the release site are likely to encounter a lower transmitter concentration during a synaptic event (Diamond, 2001; Chen and Diamond, 2002). Hence, the low-affinity competitive antagonists are progressively more effective at receptors facing low transmitter concentrations through spillover (Overstreet and Westbrook, 2003; Szabadics et al., 2007; Balakrishnan et al., 2009; Tang and Lu, 2012). Bath application of a high concentration of SR 95531 (200 μM), employed as a low affinity antagonist of GlyR (Wang and Slaughter, 2005; Beato, 2008; Balakrishnan et al., 2009), reduced the IPSC amplitudes considerably and accelerated the weighted decay time constant of single and repetitive IPSCs (Figures 4A–C; *n* = 6, RM ANOVA). Low concentrations of SR 95531 (10 and 20 μM) specifically blocked GABA_A_ receptors on SBCs and reduced the IPSC amplitudes by 18 ± 2% and 16 ± 3%, respectively (Nerlich et al., 2014). At the concentration of 200 μM, a 76 ± 5% reduction of the IPSC amplitude was observed (Figure 4D; 200 μM vs. 10 μM (*n* = 6), *p* < 0.001; 200 μM (*n* = 6) vs. 20 μM (*n* = 11), *p* < 0.001, ANOVA). This result is consistent with an additional antagonistic action of a high SR 95531 concentration at GlyR, as opposed to specific GABA_A_R blockade at concentrations of 10 and 20 μM (Nerlich et al., 2014). To further confirm the competitive antagonistic action of SR 95531 at GlyR, the glycine concentration in the cleft was increased by co-applying the glycine re-uptake inhibitor ORG 25543. This partially reversed the long τ_wd_ confirming that the current decay is regulated by the amount of available glycine (Figure 4D; change of τ_wd_: +20 μM SR = −8.1 ± 2.4%, *n* = 13; +200 μM S*R* = −35.3 ± 2.5%, *n* = 6; +200 μM SR+ORG = −21.2 ± 5.5%, *n* = 6; 20 μM vs. 200 μM SR *p* < 0.001, 200 μM SR vs. 200 μM SR+ORG *p* = 0.01, ANOVA). As the uptake blockers reveal transmitter spillover by increasing its quantity and enabling distant receptors to encounter a lower concentration of an agonist (Chen and Diamond, 2002; Thomas et al., 2011), our data showing a decay time prolongation under ORG 25543 are consistent with glycine spillover.

Inhibitory currents in SBCs show an activity-dependent depression of the IPSC peak amplitudes (I*_peak_*, Figure 4E). Yet, under the low affinity GlyR antagonist (200 μM SR 95531), the peak amplitudes showed a weaker depression which in some cells even changed into facilitation (Figures 4E,F). This result is consistent with a relief from receptor saturation and/or desensitization (Chanda and Xu-Friedman, 2010). Thus, the peak IPSC depression at high input rates is presumably caused by postsynaptic receptor saturation and/or desensitization, rather than presynaptic short-term plasticity.

Contrary to glycine signaling, GABA is apparently not activating nearby synapses. Even at high stimulation rates, the low affinity GABA_A_R antagonist TPMPA (Szabadics et al., 2007; Tang and Lu, 2012) neither affected the IPSC decay nor the amplitudes, suggesting that the postsynaptic GABA_A_R could be tightly coupled to release sites of the transmitter and, thus, encounter consistently high GABA concentration (Figures 4G–I; control vs. TPMPA τ_wd_: single *p* = 0.97; 100 Hz 10th IPSC *p* = 0.62; IPSC amplitude: single *p* = 0.67; 100 Hz 10th IPSC *p* = 0.19; *n* = 8, RM ANOVA). In summary, the apparently low uptake capacity of glycine and GABA transporters slows the decay of IPSCs, thereby enabling spillover of glycine. While we found no evidence for spillover of GABA, the transmitter quantity and intrinsic receptor properties probably account for the slow GABAergic transmission.

### Activity-dependent asynchronous release

A further mechanism putatively contributing to IPSC decay time in SBCs could be an activity-induced desynchronization of vesicle release, as shown for several inhibitory synapses (Lu and Trussell, 2000; Hefft and Jonas, 2005; Tang and Lu, 2012). To test this hypothesis, high frequency stimulation (100 and 333 Hz) of inhibitory inputs to SBCs was employed to evoke small asynchronous events in the decay phase following the IPSC trains (Figure 5A). The quantity of asynchronous IPSCs increased with stimulus duration (Figures 5B,E) and frequency (Figures 5C,F). To compare the spontaneously occurring IPSCs (sIPSC, without input stimulation) and delayed IPSCs following repetitive input stimulation, the events were detected during 1 s before synaptic stimulation and 2 s after the last evoked IPSC (see methods). The number of asynchronous events increased significantly after the 333 Hz stimulation (Figure 5E summary data for 4 cells and respective presentation of 10, 25 and 50 stimuli at 333 Hz *z* values > 10.1, *p* < 0.001, *z*-test), compared to the rate of sIPSC before stimulation (Figure 5D; mean # of sIPSCs/20 ms bin = 0.56 ± 0.08, *n* = 4). This result suggests the occurrence of activity induced asynchronous events in the decay phase of IPSC trains. Detailed analyses revealed the time course of incidence of asynchronous events which was best fit with a mono-exponential function relaxing back to the resting state. The following parameters were used to quantify the delayed release: initial amplitude (peak incidence of asynchronous events (A), an exponential time constant (τ) and a steady state value (c) (Figure 5E; fit comparison 10 vs. 25 vs. 50 stimuli *p* < 0.001, F-test). The peak incidence of asynchronous events after stimulation at 333 Hz increased almost 2-fold by extending the train from 10 to 50 pulses (A_#eIPSC_ 95% CI [lower, upper]: A_10_ = 10.6 [9.1, 12.2] events, A_25_ = 12.9 [11.7, 14.0] events, A_50_ = 18.8 [17.3, 20.3] events, 10 vs. 25 vs. 50 stimuli *p* < 0.05). The exponential time constant was also prolonged (τ_#eIPSC_ 95% CI [lower, upper]: τ_10_ = 26.5 [20.5, 37.4] ms, τ_25_ = 54.1 [46.8, 64.4] ms, τ_50_ = 79.0 [69.7, 91.1] ms, 10 vs. 25 vs. 50 stimuli *p* < 0.05), whereas the steady state values were similar to the mean sIPSC levels 1 s before stimulation (c_# eIPSC_ 95% CI [lower, upper]: c_10_ = 0.63 [0.52, 0.75] events/bin, c_25_ = 0.43 [0.33, 0.53] events/bin, c_50_ = 0.73 [0.59, 0.87] events/bin; sIPSCs ± SEM: prior to 10 eIPSCs = 0.54 ± 0.12 sIPSCs/bin, prior to 25 eIPSCs = 0.48 ± 0.10 sIPSCs/bin, prior to 50 eIPSCs = 0.74 ± 0.10 sIPSCs/bin, *p* > 0.05).

The rate of delayed release was not only dependent on the stimulus duration, but also on the stimulation frequency (Figures 5C,F summary data for 6 cells). Figure 5F, shows that presynaptic activity determines the quantity and duration of asynchronous release (A or τ, 95% CI [lower, upper]: A_100*Hz*_ = 12.4 [10.8, 13.9] events, A_333*Hz*_ = 28.3 [26.0, 30.5] events, *p* < 0.05; τ_100*Hz*_ = 58.6 [48.3, 74.7] ms, τ_333*Hz*_ = 78.5 [69.3, 90.7] ms, *p* < 0.05, *n* = 6, fit comparison 100 Hz vs. 333 Hz *p* < 0.001, *F*-test).

**Figure 6:**
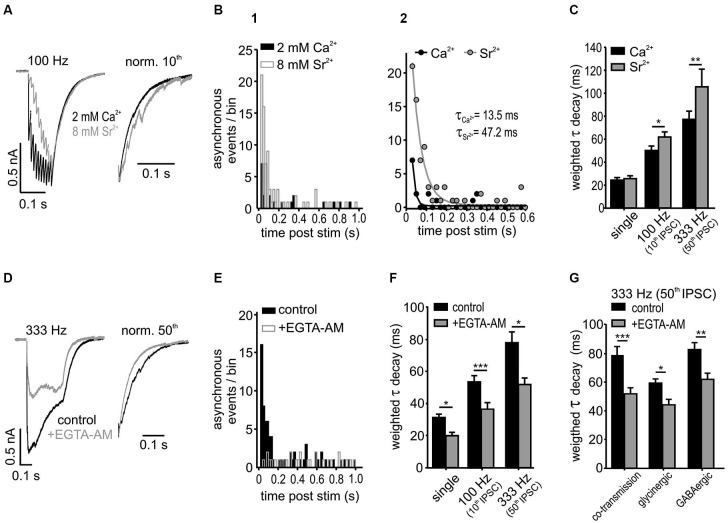
**Asynchronous release influences IPSC decay in SBCs. (A)** Superimposed responses to a 100 Hz stimulation in 2 mM calcium- and 8 mM strontium-ACSF. Right: Normalized last IPSCs showing an increased incidence of asynchronous release events under strontium. **(B1)** Summary histograms of asynchronous release events, before and after a 10 pulse stimulation at 100 Hz for both conditions (*n* = 6). **(B2)** Mono-exponential fits to the data in B1. The amount of asynchronous events after stimulation was increased and the time of increased incidence prolonged under 8 mM Sr^2+^ (A and τ: Ca^2+^ vs. Sr^2+^ *p* < 0.05). **(C)** Substitution of Ca^2+^ by Sr^2+^ prolonged the weighted decay time of IPSCs after 100 Hz and 333 Hz stimulation, but not of single IPSCs (*n* = 6, single: *p* = 0.8, 100 Hz: *p* < 0.05, 333 Hz: *p* < 0.01, RM ANOVA). **(D)** Averaged trains of 50 IPSCs at 333 Hz before and after bath application of 100 μM EGTA-AM. Right: normalized last IPSCs. **(E)** Summary histograms of asynchronous release events before and after application of EGTA-AM. Note the lack of the increase in asynchronous release after stimulation in EGTA-AM (*n* = 5, bin width: 20 ms; control: *z* = 29.2 *p* < 0.001; +EGTA-AM: *z* = −1.01 *p* = 0.16, *z*-test). **(F)** EGTA-AM shortened τ_wd_ by about 30 % regardless of stimulation frequency (*n* = 5, *p < 0.05, ***p < 0.001, RM ANOVA). **(G)** EGTA-AM equally affects the control (mixed glycine/GABA) IPSCs, isolated glycinergic (+20 μM SR 95531), and GABAergic IPSCs (+0.5 μM strychnine) in terms of decreasing the weighted decay time constants (co-transmission: *n* = 5, *p* < 0.001; glycinergic: *n* = 5, *p* < 0.05; GABAergic: *n* = 5, *p* < 0.01; ANOVA).

The putative cause for the delayed asynchronous transmitter release is the accumulation of presynaptic calcium during high frequency IPSC trains. This can be experimentally enhanced by replacing the extracellular calcium with strontium, or alternatively, the effect can be reduced by applying a cell permeable calcium chelator that accelerates the decay of presynaptic calcium transients (Goda and Stevens, 1994; Atluri and Regehr, 1996, 1998; Lu and Trussell, 2000; Xu-Friedman and Regehr, 2000; Hefft and Jonas, 2005; Best and Regehr, 2009; Tang and Lu, 2012). To test whether the delayed asynchronous events can prolong the IPSCs decay times, their incidence was increased by replacing 2 mM extracellular calcium by 8 mM strontium (Figures 6A–C). In addition to a higher frequency of asynchronous events (A) shortly after the 100 Hz stimulation (10 eIPSCs), strontium also caused a prolongation of the time course (τ) of the last IPSC (Figures 6B,C; *n* = 6, mono-exponential fit comparison: *p* < 0.001, *F*-Test; A or τ 95 % CI [lower, upper]: A_Ca_^2+^ = 6.8 [5.7, 7.9] events, A_Sr_^2+^ = 21.2 [19.4, 23.0] events; τ_Ca_^2+^ = 13.5 [9.4, 23.9] ms, τ_Sr_^2+^ = 47.2 [40.9, 55.8] ms, Ca^2+^ vs. Sr^2+^ *p* < 0.05). Consistent with the observation that asynchronous release strongly depends on synaptic activity (Figure 5), 8 mM strontium was more potent in prolonging the IPSC τ_wd_ following higher stimulation rate and longer stimulation (Figure 6C; Ca^2+^ vs. Sr^2+^ 100 Hz 10th *p* < 0.05; 333 Hz 50th *p* < 0.01, *n* = 6, RM ANOVA). Notably, single IPSC decays were not affected by the Ca^2+^ replacement (*p* = 0.8). These results suggest that the slow decay of IPSCs is indeed shaped by the asynchronous release which in turn is determined by the rate of presynaptic activity.

To further address this issue, the contribution of the delayed vesicle release to IPSC decay times was determined through reduction of presynaptic calcium with the calcium chelator EGTA-AM (100 μM) (Figures 6D–G). The IPSC amplitudes strongly decreased under EGTA-AM by 55 ± 8% for single IPSC, 71 ± 4% for the 10th IPSC at 100 Hz and 55 ± 10% for 50th IPSC at 333 Hz, presumably due to a reduction in the peak Ca^2+^ concentration in the presynaptic terminal (Atluri and Regehr, 1996). The significant increase of asynchronous events observed in control condition after 333 Hz stimulation (50 IPSCs) vanished completely under EGTA-AM (Figure 6E; change of asynchronous events after stimulation compared to baseline level before stimulation: control *z* = 29.2, *p* < 0.001; +EGTA-AM *z* = −1.01, *p* = 0.16, *z*-test). Moreover, the weighted decay time constant was shortened by 30 ± 3% regardless of stimulus condition (Figure 6F; EGTA-AM effect vs. frequency *p* = 0.9, *n* = 5, RM ANOVA) and transmitter type (glycine, GABA or both) (Figure 6G; EGTA-AM effect vs. transmission type *p* = 0.59, *n* = 5 for each condition, ANOVA). Together, these data suggest that both glycine and GABA contribute to the delayed release and thereby shape the inhibitory current profile in an activity-dependent manner.

## Discussion

The mixed glycine-GABA transmission observed in SBCs is for the most part dominated by glycine but it still exhibits slow synaptic decays resulting in a long lasting, tonic-like inhibition. While glycine primarily contributes to the IPSC amplitudes, GABA enhances and prolongs the total inhibitory conductance, especially at higher firing rates (Nerlich et al., 2014). Here we demonstrate that the resulting IPSC decay strongly depends on both the synaptic activity and the number of recruited inhibitory fibers, each increasing the amount of transmitters released. The slow glycine and GABA clearance permits a longer availability of the transmitters in the synaptic cleft, thereby enabling transmitter rebinding and glycine spillover. Moreover, the IPSC kinetics is dynamically shaped by the delayed release of glycine and GABA. In addition to the shift towards slower GABAergic transmission at higher input rates (Nerlich et al., 2014), these activity-dependent mechanisms jointly determine the remarkably slow inhibition in SBCs.

### Transmitter rebinding and spillover contribute to the IPSC decay

The SBCs of the cochlear nucleus are the first synaptic center where primary auditory input from the cochlea is integrated with a higher-order, acoustically-evoked inhibition to preserve or improve temporal precision on the sub-millisecond scale (Gai and Carney, 2008; Dehmel et al., 2010; Kuenzel et al., 2011). Such synaptic inhibition is likely to contribute to the temporal synchronization of AP discharges to a particular phase of a low frequency tone burst (Joris et al., 1994; Paolini et al., 2001; Dehmel et al., 2010). Saying this, it should not be disregarded that also other previously discussed mechanisms (Nerlich et al., 2014), such as coincidence of presynaptic inputs also add to the synchronicity of postsynaptic SBC discharges (Kuhlmann et al., 2002; Xu-Friedman and Regehr, 2005). With respect to the inhibitory effects, it was shown that an activity-dependent regulation of inhibitory strength mediated trough a slow glycine-GABA transmission adjusts the fidelity at the endbulb of Held synapse towards fast rising and large EPSPs (Kuenzel et al., 2011; Xie and Manis, 2013; Nerlich et al., 2014). The present data further demonstrate that the inhibitory strength crucially depends on the presynaptic firing rate, which is of particular physiological importance given the weak inhibition at lower sound intensities, i.e., low firing rates (Kuenzel et al., 2011). Following strong inhibitory stimulation, slow glycine and GABA clearance from the synaptic cleft appears to enable ongoing rebinding of transmitters and intersynaptic pooling of glycine. A similar mechanism was previously shown to mediate prolonged inhibition of granule cells in the rat DCN (Balakrishnan et al., 2009) and of the nucleus laminaris neurons in the chick auditory brainstem (Tang and Lu, 2012). Several lines of evidence suggest that both the GlyT2 and the GABA transporters GAT1/3 contribute to the kinetics of activity-dependent inhibition at SBCs. Particularly, the slow decay phase (τ_slow_) was affected by increasing stimulus frequencies, suggesting longer availability of transmitters. Similar τ_slow_ prolongation was also observed in the presence of glycine and GABA transporter antagonists. As the respective slowly decaying current is most likely due to slow transmitter clearance (Otis et al., 1996; Williams et al., 1998), our data are consistent with transporter saturation during higher neuronal activity. In line with this, the vesicular inhibitory amino acid transporter (VGAT), which depends on the supply of cytosolic transmitter to enable synaptic corelease of glycine and GABA (Wojcik et al., 2006), is apparently not the rate limiting factor for efficient refilling of inhibitory vesicles (Apostolides and Trussell, 2013).

At mixed glycine-GABA synapses, several factors determine the characteristics of the postsynaptic response: (i) the glycine/GABA ratio in synaptic vesicles which is not only determined by the higher VGAT affinity for glycine than for GABA (McIntire et al., 1997; Bedet et al., 2000); but also crucially depends on the availability of glycine through GlyT2 activity (Rousseau et al., 2008); and (ii) the postsynaptic receptor expression levels, clustering, and localizations (Todd et al., 1996; Dugué et al., 2005). In our recent study, we described a relative increase of the GABAergic component from 5 to 12% of the mixed IPSC amplitude during ongoing synaptic activity (Nerlich et al., 2014). When glycine and GABA were puff-applied (equimolar concentrations of 500 μM), the current amplitude ratio was ~3:2 (1.7:1.2 nA), indicating that GABA_A_R availability is probably not the rate limiting factor for restricted GABAergic contribution to the IPSC. Presently, we found no evidence for GABA spillover to distant receptors that could account for the activity-dependent increase in the GABAergic proportion. Thus, the slow glycine clearance by GlyT2 and its low intracellular availability may possibly explain the progressively higher GABAergic signaling at *in vivo*-like firing rates.

Compared to the events elicited in 1.2 mM [Ca^2+^]*_o_*, IPSCs measured under the standard 2 mM [Ca^2+^]*_o_* condition were found to be slower after shorter stimulus trains or at lower input rates, possibly indicating a contribution of multivesicular release. However, longer or high frequency stimulation (50 pulses at 100 Hz, 10 or 50 pulses at 333 Hz) evoked IPSC of comparable amplitudes towards the end of the train and the similar decay kinetics of the last event. This suggests that prolonged neuronal activity at physiological-like rates leads to a steady state transmitter level in the cleft, independent of the initial release probability. The data also argue against the prominent receptor desensitization, because its effect would render IPSCs faster with increasing transmitter concentrations at higher rates (Jones and Westbrook, 1996), which was not observed in our experiments.

Glycine receptor saturation and spillover onto nearby or distant receptors probably shapes the IPSC kinetics at physiologically relevant firing rates. Due to the lack of effect of the low-affinity GABA_A_ antagonist, we conclude that the respective receptors are probably tightly coupled to the release sites and unlikely to saturate. In line with this notion, is the lack of GABA_A_ α6 and δ subunit expression on SBCs (Campos et al., 2001) which were shown to constitute the extrasynaptic receptors (Nusser et al., 1998; Kullmann et al., 2005). The slow IPSC kinetics measured at SBCs contrast with respective data for other inhibitory auditory synapses with decay time constants <5 ms, in which GABA is either not engaged or plays a minor role in determining the response decay time (Awatramani et al., 2004; Magnusson et al., 2005; Chirila et al., 2007; Couchman et al., 2010). As the SBC IPSCs progressively resembled the kinetics of pharmacologically isolated GABAergic events at increasing input frequencies, the mechanisms engaged with GABA release are likely to regulate the duration of inhibition at physiologically relevant rates (Nerlich et al., 2014). One possible contributing mechanism, though not a focus of the present study, is the kinetics of the GABA_A_ receptor itself. The α3 subunit, linked to slow kinetics, deactivation- and desensitization rates (Verdoorn, 1994; Gingrich et al., 1995) is coexpressed with the subunits α1, α5, β3 and γ2L in the rat AVCN (Campos et al., 2001). In olfactory bulb neurons of juvenile rats, the deactivation kinetics of GABA-evoked mIPSCs can span a τ_wd_ range of 3–30 ms through the expression of different compositions of the fast α1 and the slow α3 subunits in the receptor heteromers (Eyre et al., 2012). Therefore, it is conceivable that the specific composition of GABA_A_ subunits in SBCs partially contributes to an increase in τ_wd_ which ranged between 20–90 ms in an activity-dependent manner. Notably, even the isolated glycinergic spontaneous- and evoked-IPSCs exhibit slow synaptic decays (Xie and Manis, 2013, 2014; Nerlich et al., 2014). This may be surprising, given the general developmental down-regulation of the GlyR α2 subunit, associated with slower receptor kinetics (Veruki et al., 2007) in the cochlear nucleus and in the superior olivary compex (Sato et al., 1995; Friauf et al., 1997). However, the developmental replacement by the α1 subunit in the AVCN seems in the AVCN, as low levels of α2 mRNA were found up to the third postnatal week (Piechotta et al., 2001). Our data substantiate the hypothesis of a persistent α2 subunit expression throughout adulthood by showing that single pulse stimulation evokes IPSCs of similar τ_wd_ for pharmacologically isolated glycinergic, GABAergic, and mixed glycine-GABA events (Nerlich et al., 2014). Here, further corroboration is provided by showing comparable slow kinetics of the spontaneous IPSCs and IPSCs synaptically elicited in low release probability conditions. In both cases, the amount of released transmitters is presumed low, thus reducing the contribution of activity-dependent mechanisms. Although our results argue for a role of slow GlyR and GABA_A_R, this still seems to be only an additional mechanism shaping the overall kinetic profile.

### Inhibitory kinetics is shaped by the activity-induced delayed release

Asynchronous (or delayed) transmitter release is another mechanism to generate a long-long lasting inhibition in the brain (Lu and Trussell, 2000; Hefft and Jonas, 2005; Tang and Lu, 2012). The ongoing synaptic activity can lead to Ca^2+^ accumulation in the presynaptic terminal, thereby causing the loss of coupling between the presynaptic AP and the release machinery (Chen and Regehr, 1999). Our data from the present and previous study (Nerlich et al., 2014) consistently show the activity-dependent change of release mode. The individual IPSCs were clearly segregated at 100 Hz, indicating synchronized quantal release, whereas the 333 Hz stimulation evoked a large plateau current caused by asynchronous release. Notably, the delayed asynchronous release events, mediated by both glycine and GABA, were observed at both frequencies during the decay phase of the last IPSC. The prominent effect of the slow calcium chelator EGTA-AM in our experiments, suggests a loose coupling between presynaptic Ca^2+^ channels and the Ca^2+^ sensor, possibly in a form of microdomains (Meinrenken et al., 2002; Eggermann et al., 2012; Vyleta and Jonas, 2014). The Ca^2+^ dependance of release machinery was, however, not in focus of the present study. Alongside transmitter rebinding and spillover, asynchronous release is a major mechanism contributing to activity-dependent changes in inhibitory duration.

### Efficacy of slow inhibition

Neurons in the auditory brainstem generate APs with an extraordinary temporal accuracy required for the computation of sound source location (for review see, Grothe et al., 2010). In the binaural nuclei of the superior olivary complex, the fast and mainly glycinergic inhibition may contribute by providing high precision phasic suppression of excitatory conductance (Awatramani et al., 2004; Magnusson et al., 2005; Chirila et al., 2007; Couchman et al., 2010; Roberts et al., 2013, 2014; Myoga et al., 2014). The monaural neurons in the mammalian cochlear nucleus, such as bushy cells of the AVCN and the granule cells of the DCN, on the other hand, utilize slow inhibition characterized by an activity-dependent conductance build-up (Balakrishnan et al., 2009; Xie and Manis, 2013). *In vivo*, the onset of acoustically evoked inhibition on SBCs is delayed compared to the excitation (Kuenzel et al., 2011; Nerlich et al., 2014) which cannot be solely explained by the polysynaptic inhibitory pathway causing a delay of just a few milliseconds (Smith and Rhode, 1989; Wickesberg and Oertel, 1990; Saint Marie et al., 1991; Ostapoff et al., 1997; Campagnola and Manis, 2014). Rather, the new data suggest that the slow time course of inhibition is predominantly determined by the receptor kinetics and transmitter clearance during short stimuli, whereas long duration or high frequency stimulation additionally engage spillover of glycine and asynchronous release of both glycine and GABA to prolong IPSCs. The contribution of these synaptic mechanisms can explain the slow onset of inhibition observed *in vivo*. With increasing sound intensity, the dynamic adjustment of inhibitory potency through synergistic glycine-GABA signaling (Nerlich et al., 2014) would ensure effective integration with strong and coincident excitatory inputs leading to non-monotonic rate-level functions and improved temporal precision of the SBC output (Kopp-Scheinpflug et al., 2002; Dehmel et al., 2010; Kuenzel et al., 2011). Consistent with this hypothesis, a modeling study of SBC inhibition with fast IPSC kinetics as those measured in AVCN T-stellate cells impaired the temporal precision of spiking (Xie and Manis, 2013). Thus, the activity-dependance of slow inhibition seems to be a critical factor for precise temporal processing in SBCs.

## Conflict of interest statement

The authors declare that the research was conducted in the absence of any commercial or financial relationships that could be construed as a potential conflict of interest.
